# Immunonutritional Markers and the Protective Role of Sternal Irrigation and Antibiotic-Impregnated Membranes in Sternal Wound Infection: A Retrospective Cohort Study

**DOI:** 10.3390/life15081163

**Published:** 2025-07-23

**Authors:** Ebubekir Sönmez, İzatullah Jalalzai, Ümit Arslan, Alperen Yıldız, Furkan Çelik, Merve Çetin

**Affiliations:** 1 Department of Cardiovascular Surgery, Faculty of Medicine, Atatürk University, Erzurum 25030, Türkiye; e.sonmezkvc@gmail.com (E.S.); ijalalzai@gmail.com (İ.J.); ua.alpera@gmail.com (A.Y.); frkn462525@gmail.com (F.Ç.); 2 Department of Pharmacognosy, Faculty of Pharmacy, Bülent Ecevit University, Zonguldak 67100, Türkiye; mervecetin25@hotmail.com

**Keywords:** sternal wound infection, sternotomy, local antibiotic prophylaxis, collagen membrane, CRP, neutrophil-to-lymphocyte ratio, CRP-to-albumin ratio, prognostic nutritional index, inflammation, biomarkers

## Abstract

Background: Sternal wound infections (SWIs) remain a significant complication following cardiac surgery. Inflammatory and nutritional status are increasingly recognized as key contributors to their development. This study aimed to investigate the predictive utility of immunonutritional biomarkers and to evaluate the protective effect of combining sternal irrigation with an antibiotic-impregnated membrane. Methods: This retrospective cohort study included 480 patients undergoing off-pump coronary artery bypass grafting. Patients were categorized based on sternal management strategy (standard closure or local prophylaxis using gentamicin-enriched irrigation combined with an antibiotic-impregnated fascia lata membrane) and according to the severity of SWIs, classified as superficial or deep. Inflammatory and nutritional markers—including C-reactive protein (CRP), neutrophils, lymphocytes, albumin, neutrophil-to-lymphocyte ratio (NLR), C-reactive protein-to-albumin ratio (CAR), and prognostic nutritional index (PNI)—were assessed at three time points: preoperatively, on postoperative day 3, and after week 1. Results: SWIs were observed in 93 patients, including 75 superficial and 18 deep infections. The combined prophylactic approach was associated with a nearly 1.8-fold reduction in deep SWIs (OR: 0.55; 95% CI: 0.15–0.87) and a modest reduction in superficial infections (OR: 0.89; 95% CI: 0.5–1.3; *p* = 0.061). Threshold values of 3.75 for preoperative NLR, 9.8 for ΔNLR, and 16.7 for ΔCAR demonstrated strong predictive capacity for identifying patients at increased risk of developing deep SWIs. Patients receiving local prophylaxis exhibited significantly lower CRP, NLR, and CAR values and higher PNI levels at all time points. Conclusions: The combination of sternal irrigation and local antibiotic prophylaxis appears to confer protection against SWIs, potentially by mitigating postoperative inflammation. Immunonutritional biomarkers offer a promising means for early risk stratification. To confirm their clinical utility and broader applicability, these results should be validated in prospective, multicenter studies encompassing a wider range of cardiac surgical procedures.

## 1. Introduction

Although median sternotomy is classified as a clean surgical procedure, sternal wound infections (SWIs) remain a significant postoperative complication, driven by a multifactorial interplay of patient- and procedure-related risk factors. The clinical burden of SWIs is considerable—not only due to their prolonged and often recurrent course, but also because they are associated with mortality rates approaching 30–50%. Consequently, preventing SWIs has become a critical focus in modern cardiothoracic surgery [1,2,3]. While specific risk factors for SWIs vary depending on patient characteristics and surgical techniques, the frequent isolation of skin flora and both Gram-positive and Gram-negative bacteria from infected sites highlights the need for strict aseptic techniques, optimized sternal closure methods, and precisely targeted antibiotic prophylaxis [4,5,6]. In addition to conventional intravenous antibiotic administration, adjunctive intraoperative strategies—particularly the local application of high-concentration antibiotics before sternal closure—have gained interest as potential measures to further mitigate infection risk [7,8].

A meta-analysis by Kowalewski et al. [9] demonstrated that gentamicin-impregnated collagen sponges can reduce the incidence of SWIs by approximately 40%. However, Konishi et al. [10] emphasized that comprehensive, multimodal strategies—encompassing preoperative microbial surveillance and technical refinements in surgical practice—may offer superior preventive efficacy. Within this framework, identifying individual risk profiles and implementing structured risk assessment models are crucial for early stratification and timely preventive interventions [11,12,13]. Furthermore, increasing attention has been directed toward composite indices that incorporate inflammatory cell ratios and nutritional markers, which have recently gained recognition as promising predictors of adverse postoperative outcomes [14,15].

Among the emerging biomarkers, the C-reactive protein-to-albumin ratio (CAR) and the neutrophil-to-lymphocyte ratio (NLR) have been extensively studied as indicators of systemic inflammation. Yet their precise role in the pathogenesis of SWIs remains to be fully elucidated. Parla et al. [16] reported significantly elevated NLR values in patients who developed SWIs and identified a high NLR as an independent risk factor for infection. Additionally, nutritional status has garnered increasing interest due to its capacity to modulate inflammatory cascades and compromise tissue regeneration, thereby exerting a pivotal influence on wound healing [17]. The prognostic nutritional index (PNI), a validated surrogate for nutritional reserve, has also shown prognostic value in cardiovascular cohorts [18]. In individuals with coronary artery disease, lower PNI scores have been associated with a nearly 1.7-fold increase in all-cause mortality and a 1.6-fold higher incidence of major adverse cardiac events [19].

This study aimed to assess the efficacy of intraoperative sternal irrigation using an antibiotic solution and the application of a locally delivered antibiotic-impregnated membrane in reducing SWI occurrence following off-pump coronary artery bypass grafting (OPCABG). A secondary objective was to investigate whether selected inflammatory and nutritional markers serve as independent and clinically relevant predictors of postoperative infection.

## 2. Materials and Methods

### 2.1. Study Design and Patient Selection

This retrospective analytical cohort study included 480 patients who underwent OPCABG between 2017 and 2024 at a single tertiary cardiovascular center. All procedures were performed by the same surgical team. Of the approximately 640 patients initially evaluated, several were excluded based on predefined criteria. These included patients with missing laboratory data (*n* = 45), those with chronic kidney disease requiring dialysis (*n* = 25), and others who underwent minimally invasive coronary artery by-pass procedures (*n* = 14), redo operations (*n* = 13), or surgery following failed percutaneous coronary intervention (*n* = 8). Additional exclusions were cases treated with sternal irrigation alone without membrane placement (*n* = 25), patients requiring intra-aortic balloon pump support (*n* = 10), those with traumatic coronary injury (*n* = 5), malignancy (*n* = 4), prior liver or kidney transplantation (*n* = 3), use of immunosuppressive agents (*n* = 4), operations performed under high spinal anesthesia (*n* = 3), and one case with a porcelain aorta (*n* = 1).

In 2021, a revised institutional protocol was introduced, incorporating intraoperative sternal irrigation with gentamicin-enriched saline and application of an antibiotic-impregnated fascia lata membrane during sternal closure. Accordingly, patients were categorized into two groups based on the surgical period: the local antibiotic (LA) group (*n* = 230, operated between 2021 and 2024) and the control group (CG) (*n* = 250, operated between 2017 and 2021, prior to protocol implementation).

Demographic and clinical data were retrieved from a systematically maintained hospital database composed of prospectively recorded physician notes and administrative records. Body mass index (BMI) was calculated by dividing weight (kg) by the square of height (m^2^). Inflammatory and nutritional indices—including the CAR and PNI—were calculated from routine laboratory values using previously validated formulas [20], as outlined in Table 1. To evaluate perioperative dynamics, delta values (Δ) were calculated as the difference between preoperative values and those obtained on postoperative day 3.

To ensure a uniform assessment of the inflammatory response, only patients who underwent OPCABG without cardiopulmonary bypass (CPB) were included [21]. Inflammatory markers were assessed at three time points: preoperatively, on postoperative day 3, and at the end of the first postoperative week. Based on clinical and microbiological criteria, patients who developed SWIs were categorized into superficial and deep infection subgroups [22]. Although we did not apply a strict time-based classification, SWIs were categorized according to the depth of tissue involvement and the need for surgical intervention. Superficial infections included wounds with persistent drainage or fat necrosis that did not require sternal reopening, as well as any subcutaneous discharge observed at any postoperative time, including wire-related reactions. Deep SWIs were defined as purulent sternal wounds necessitating surgical revision—such as debridement or flap reconstruction—even in the absence of mediastinal contamination [23].

The diagnosis of SWIs was established clinically by the surgical team, based on local signs including erythema, tenderness, purulent discharge, and wound dehiscence. In patients with suspected infection, both wound and blood cultures were routinely obtained to identify causative pathogens and guide antimicrobial therapy. Classification into superficial or deep infection types was made in accordance with institutional guidelines and supported by microbiological evidence when available.

This study aimed to determine whether intraoperative sternal interventions—specifically, antibiotic irrigation and membrane application—conferred a protective effect against SWIs and modulated the postoperative systemic inflammatory response. Furthermore, the association between immunonutritional indices and infection severity was analyzed, with particular emphasis on their predictive value and the identification of clinically relevant cutoff thresholds.

### 2.2. Inclusion Criteria

- Age ≥ 18 years.
- Undergoing isolated off-pump coronary artery bypass grafting (OPCABG) via median sternotomy.
- Availability of complete laboratory data at all four predefined time points (preoperative, postoperative day 1, day 3, and day 7).
- Regular postoperative outpatient follow-up attendance.

### 2.3. Exclusion Criteria

To minimize confounding factors and enhance the interpretability of systemic inflammatory markers, the following exclusion criteria were applied:

- Patients with incomplete laboratory or clinical data, including those who died or were lost to follow-up within the first postoperative week, or who failed to attend scheduled outpatient follow-up visits after discharge.
- Patients in whom cardiopulmonary bypass was used during the procedure.
- Minimally invasive coronary artery bypass techniques.
- Acute myocardial infarction within the preceding 30 days.
- Emergent surgical indication or the need for intra-aortic balloon pump support.
- Redo coronary artery bypass procedures.
- Dialysis-dependent chronic kidney disease.
- Clinically diagnosed hematological, immunological, or autoimmune disorders.
- The use of chemotherapy or immunosuppressive medication.

### 2.4. Surgical Technique

Preoperative hair removal was performed with electric clippers one day prior to surgery, followed by antiseptic cleansing using a chlorhexidine solution. In the operating room, skin preparation was completed with the application of 10% povidone-iodine solution (Batticon^®^). Perioperative antibiotic prophylaxis adhered to institutional standards, consisting of intravenous cefazolin (2 g), initiated within 60 min before skin incision and maintained for 48 h postoperatively [24]. All procedures were conducted via median sternotomy. The number of distal anastomoses was recorded for each patient. BITA grafting was employed in cases requiring full arterial revascularization, particularly among non-obese, non-diabetic patients undergoing on-pump coronary artery bypass grafting. However, no patients in the present off-pump cohort received BITA grafts. All procedures involved either a single left internal thoracic artery (LITA) graft or a radial artery graft; in patients for whom the radial artery was not suitable, a saphenous vein graft was used instead. Operative time was defined as the interval between the initial skin incision and the final closure of the skin and subcutaneous tissues, including sternal closure (from skin incision to skin closure).

**Figure 1:**
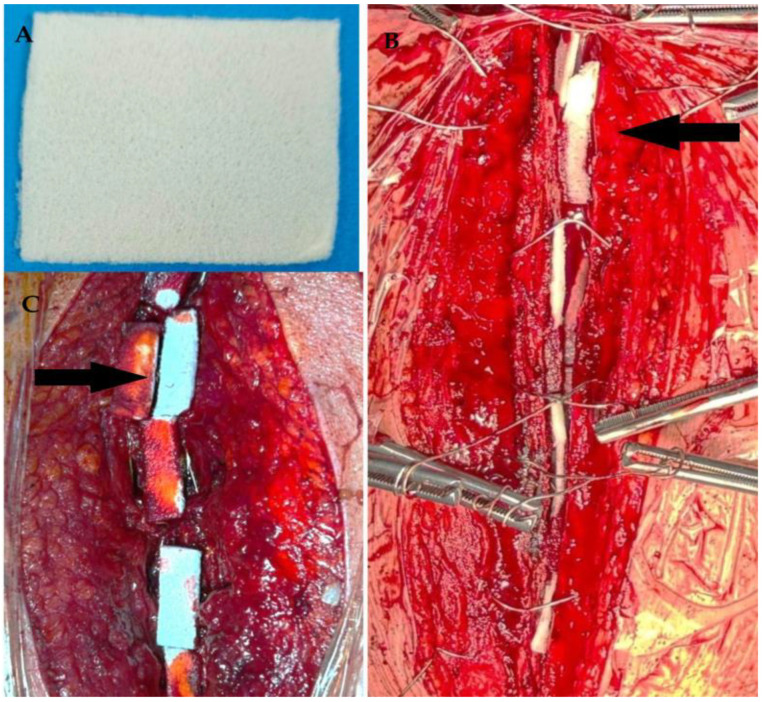
Intraoperative application of antibiotic-loaded collagen membrane. (**A**) Antibiotic-impregnated collagen membrane trimmed to 5 × 5 cm for intraoperative use. (**B**) Placement of membrane between sternal edges prior to closure. (**C**) Application of membrane both anterior and posterior to sternum in obese female patient (black arrows indicate trimmed portion of membrane).

In the intervention group, sternal closure included the application of an 80 × 200 mm ReadiGraft^®^ Fascia Lata patch—an absorbable allograft derived from human fascia lata (LifeNet Health, Virginia Beach, VA, USA)—soaked in 500 mL of isotonic saline (0.9% NaCl) containing 240 mg of gentamicin (120 mg/2 mL; two ampoules). The membrane was equilibrated at room temperature for 10 min, then cut into multiple segments and placed between the sternal edges along the entire midline incision. Closure was performed using the figure-of-eight wiring technique. Following sternal approximation, the remaining antibiotic solution was applied to the overlying soft tissues via brush-assisted irrigation, and closure of the subcutaneous tissue and skin was completed with absorbable sutures [25,26]. In contrast, the control group underwent standard sternal closure using stainless-steel wire cerclage, without the use of topical antibiotic solutions or membranes (Figure 1A–C).

### 2.5. Statistical Analysis

All statistical analyses were conducted using SPSS (version 28; IBM Corp., Armonk, NY, USA), Jamovi (version 2.6.44), and G*Power (version 3.1.9.7; Heinrich Heine University, Düsseldorf, Germany). Categorical variables were expressed as frequencies and percentages, while continuous variables were presented as the mean ± standard deviation (SD) for normally distributed data, and as the median with interquartile range (IQR) for non-normally distributed data. Normality of distribution was assessed using the Kolmogorov–Smirnov and Shapiro–Wilk tests, and homogeneity of variances was evaluated with Levene’s test.

Comparisons between categorical variables were performed using the chi-square or Fisher’s exact test, as appropriate. For continuous variables, independent samples t-tests were used for normally distributed data, while the Mann–Whitney U test was applied for non-normally distributed data. In cases where the assumption of equal variances was violated, the Brunner–Munzel test was employed as a robust non-parametric alternative. To ensure robustness in non-parametric comparisons and regression models, bias-corrected and accelerated (BCa) bootstrap resampling with 5000 iterations was used to generate confidence intervals.

Prior to outcome analyses, baseline demographic and clinical characteristics were compared across groups to assess initial balance. As no significant differences were observed in key confounders (e.g., age, sex, comorbidities), the groups were deemed sufficiently comparable, and propensity score matching was not performed.

Inflammatory and nutritional formulas (CAR, NLR, and PNI) measured at three time points (preoperative, postoperative day 3, and postoperative week 1) were analyzed using repeated measures ANOVA or the Friedman test, based on data normality. Bonferroni correction was applied for post hoc analyses to adjust for multiple comparisons.

To identify variables independently associated with the development of SWIs, univariate analyses were first conducted. Variables with a *p*-value < 0.10 were subsequently included in a multivariate logistic regression model. Odds ratios (ORs) with 95% confidence intervals (CIs) were reported. All continuous predictors included in the regression model were standardized using Z-scores to enhance comparability and mitigate scale-related bias. A two-tailed *p*-value < 0.05 was considered statistically significant.

## 3. Results

A total of 480 patients (123 females and 357 males) were included in the analysis and allocated to either the control group (CG) or the local antibiotic group (LA group). The CG consisted of 54 females (mean age: 63.5 ± 7.5 years) and 196 males (mean age: 61.0 ± 8.8 years), while the LA group comprised 69 females (mean age: 62.0 ± 8.0 years) and 161 males (mean age: 62.5 ± 7.8 years). There were no significant differences between the groups in terms of sex distribution (*p* = 0.065), mean age (*p* = 0.438), or the prevalence of diabetes mellitus (*p* = 0.539), hypertension (*p* = 0.845), or current smoking (*p* = 0.181).

In the overall cohort, 106 patients were receiving treatment for chronic obstructive pulmonary disease, 47 had documented peripheral arterial disease, and 26 had a history of cerebrovascular events. Obesity, defined as a body mass index (BMI) ≥ 30 kg/m^2^, was similarly distributed between groups, with 63 cases in the CG and 55 in the LA group (*p* = 0.744). Baseline demographic and clinical characteristics are summarized in Table 2.

The number of distal anastomoses was comparable between the control and LA groups (2.8 ± 0.8 vs. 2.9 ± 0.9; *p* = 0.315), as was the volume of red blood cell transfusion (1.3 ± 0.5 vs. 1.2 ± 0.4 units; *p* = 0.850). Although the operative time was significantly longer in the control group (115 ± 37 vs. 107 ± 35 min; *p* = 0.033), this difference may be attributable to the higher prevalence of obese patients in that cohort. Notably, the implementation of sternal interventions in the LA group appears to have mitigated this discrepancy, resulting in operative durations that approximated those observed in the control group.

On both postoperative day 3 and day 7, patients in the CG demonstrated significantly higher CRP levels (*p* < 0.001 and *p* = 0.002, respectively) and neutrophil counts (*p* < 0.001 for both) compared to those in the LA group. These changes were accompanied by markedly lower lymphocyte counts (*p* < 0.001 for both), resulting in significantly elevated NLR values at both time points (*p* < 0.001). Similarly, CAR values were significantly higher on day 3 (*p* < 0.001) and at week 1 (*p* = 0.005).

The increase in CAR was driven not only by elevated CRP but also by a simultaneous decline in serum albumin levels, suggesting both intensified systemic inflammation and nutritional deterioration. In line with these findings, PNI scores were significantly lower in the CG at both time points (*p* < 0.001).

Delta analyses comparing preoperative values with those on day 3 further highlighted this trend: the CG showed greater increases in the NLR (mean difference: 3.11; partial η^2^ = 0.557; *p* < 0.001) and CAR (mean difference: 6.50; partial η^2^ = 0.849; *p* < 0.001), indicating a more pronounced postoperative inflammatory response. These results are detailed in Table 3.

**Figure 2:**
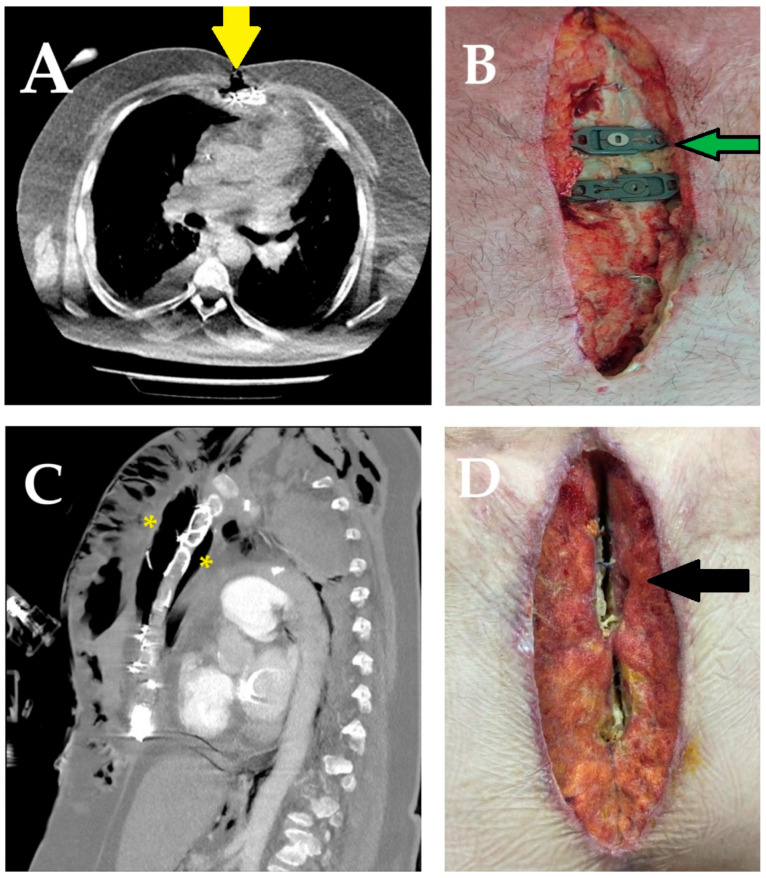
Representative cases of superficial and deep sternal wound infections. (**A**,**B**) An axial CT image of a 68-year-old obese, diabetic, and actively smoking male patient presenting with superficial wound drainage reveals subcutaneous air density anterior to the sternum (yellow arrow). During revision surgery, the incision had to be extended; the sternum and surrounding soft tissues were debrided, and a subpectoral muscle flap was used for reconstruction. The green arrow shows the sternal fixation material. (**C**,**D**) A sagittal CT view of a morbidly obese, diabetic female patient with deep sternal wound infection and dehiscence reveals extensive air accumulation both anterior and posterior to the sternum (yellow asterisks). During revision surgery, the sternum was found to be severely destructed (black arrow), and mediastinitis was treated with an omental flap.

A more detailed assessment of risk factors and laboratory parameters was conducted through subgroup analyses within both cohorts, based on the primary outcome of SWIs. In total, 93 patients (19.4%) developed SWIs, including 75 cases (15.6%) of superficial and 18 cases (3.75%) of deep infection. Compared to the control group—which recorded 49 superficial and 14 deep infections—the LA group had significantly fewer events, with only 26 superficial and 4 deep infections. Fisher’s exact test confirmed that these reductions were statistically significant for both superficial (*p* = 0.016) and deep SWIs (*p* = 0.030). To assess the statistical robustness of these findings, a post hoc power analysis was conducted based on chi-square results (Cohen’s w = 0.24). The calculated power was 88% for a total sample size of 480 at a significance level of α = 0.05, indicating sufficient sensitivity to detect meaningful differences in SWI severity between groups (Figure 2A–D).

No mortality was observed in the LA group due to deep SWIs, whereas three patients (16%) in the control group died as a result of sepsis and prolonged ventilatory support. In the control group, wound cultures frequently yielded Staphylococcus aureus, Acinetobacter spp., Pseudomonas aeruginosa, Klebsiella pneumoniae, or Enterobacter species. In contrast, among LA group patients with deep SWIs, three had coagulase-negative staphylococci, and one had Staphylococcus aureus growth. Advanced reconstructive procedures—such as omental flap transposition for mediastinitis and bilateral pectoralis major muscle advancement flaps for sternal coverage—were performed as necessary.

In superficial SWIs, the cultured organisms primarily included skin commensals, notably Enterococcus spp. and Staphylococcus epidermidis. These infections were successfully managed through local wound care, vacuum-assisted closure (VAC) therapy, minor surgical debridement, and regular dressing changes, resulting in complete clinical resolution in all cases.

Patients who developed deep SWIs were generally older, exhibited higher EuroSCORE values, and had a higher prevalence of obesity. Compared to those with superficial or no infection, they also demonstrated poorer glycemic and lipid control. Moreover, these patients underwent longer operative procedures and required greater volumes of blood transfusion.

In the postoperative period, patients with deep SWIs consistently exhibited elevated CRP levels—medians of 205.0 mg/L on postoperative day 3 and 60.0 mg/L at one week—alongside increased neutrophil counts (medians of 16.0 and 12.0 × 10^3^/μL, respectively) and reduced lymphocyte counts (medians of 0.8 and 1.0 × 10^3^/μL). These findings reflect a substantial inflammatory burden. Concurrent declines in serum albumin and lymphocyte counts further suggest compromised nutritional reserves.

Bootstrap-adjusted comparisons revealed that patients with superficial or deep SWIs in the LA group consistently showed lower postoperative levels of inflammatory markers—particularly CAR and NLR—than those in the control group. Furthermore, stratified analysis by SWI severity demonstrated that deep infections were associated with markedly greater early postoperative changes in key biomarkers. Specifically, ΔNLR and ΔCAR values were significantly higher in deep SWIs (*p* = 0.003 and *p* < 0.001, respectively), reflecting a more abrupt shift in host immune response. A comprehensive summary of clinical profiles and laboratory and perioperative findings by infection severity is provided in Table 4.

Potential predictors of SWI severity were initially identified through univariate analyses and subsequently incorporated into a multinomial logistic regression model. Using the “no infection” group as the reference category, associations with both superficial and deep sternal wound infections were examined. The model exhibited a good overall fit, with a McFadden R^2^ of 0.480 and a Nagelkerke R^2^ of 0.671, reflecting moderate to strong explanatory capacity.

Multivariate analysis revealed that the combined use of sternal irrigation and an antibiotic-impregnated membrane was associated with an approximately 1.8-fold reduction in the risk of deep SWIs (OR: 0.55; 95% CI: 0.15–0.87; *p* = 0.001). Conversely, its effect on superficial infections was less pronounced and did not reach statistical significance (OR: 0.89; 95% CI: 0.5–1.3; *p* = 0.061). Based on the ROC-derived threshold, operative procedures exceeding 108 min were associated with a progressively increased risk of deep SWIs. Specifically, for every additional 15 min increment beyond this threshold, the odds of developing deep SWIs increased by approximately 2.1-fold (OR: 2.1; 95% CI: 1.2–3.1; *p* = 0.012). Independent predictors for both superficial and deep infections included female sex, diabetes mellitus, obesity (BMI ≥ 30 kg/m^2^), and active smoking. Among laboratory parameters, elevated NLR, reduced PNI, glycated hemoglobin (HbA1c) ≥ 7%, and hypoalbuminemia (serum albumin < 3.8 g/dL) emerged as significant and independent risk factors for SWI development.

The absence of markedly elevated CRP concentrations in the preoperative period may have attenuated the diagnostic sensitivity of CRP-based indices such as the CAR. Without an active systemic inflammatory state at baseline, the CAR may be less effective in capturing the combined burden of inflammation and nutritional compromise. Consequently, CRP and albumin appeared to demonstrate greater individual predictive value when considered independently. A comprehensive summary of predictors associated with both superficial and deep SWIs, as identified in the regression model, is presented in Table 5.

**Figure 3:**
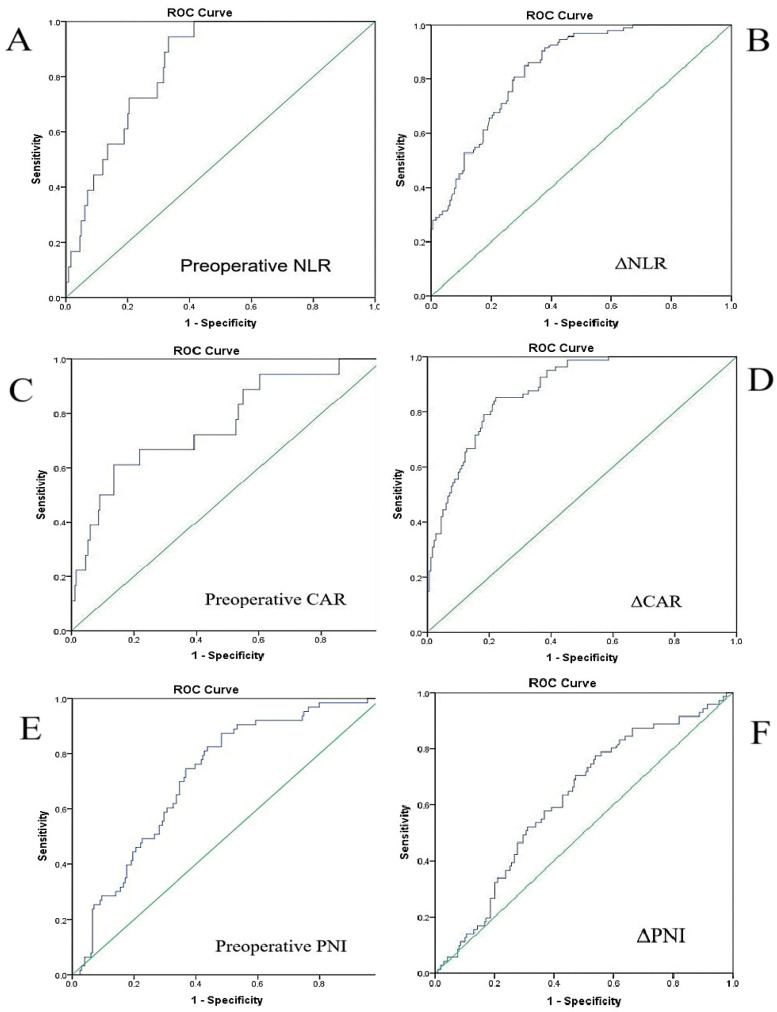
ROC curves demonstrating the predictive performance of inflammatory and nutritional markers for deep sternal wound infections. (**A**) The ROC curve of preoperative neutrophil-to-lymphocyte ratio (NLR) for predicting deep SWIs (AUC: 0.880; 95% CI: 0.82–0.93; *p* < 0.001; cutoff: 3.75). (**B**) The ROC curve of ΔNLR for deep SWIs, showing an AUC of 0.841 (95% CI: 0.77–0.90; *p* < 0.001; cutoff: 9.8). (**C**) The ROC curve of preoperative CRP-to-albumin ratio (CAR), showing moderate discriminative ability (AUC: 0.760; 95% CI: 0.64–0.89; *p* < 0.001; cutoff: 3.2). (**D**) The ROC curve of ΔCAR, demonstrating the highest overall diagnostic accuracy (AUC: 0.895; 95% CI: 0.80–0.99; *p* < 0.001; cutoff: 16.7). (**E**) The ROC curve of the preoperative prognostic nutritional index (PNI), showing fair diagnostic accuracy (AUC: 0.731; 95% CI: 0.60–0.89; *p* < 0.001; cutoff: 39.5). (**F**) The ROC curve of ΔPNI, showing limited discriminative ability (AUC: 0.619; 95% CI: 0.545–0.692; *p* = 0.003).

Receiver operating characteristic (ROC) analyses were conducted to evaluate the diagnostic performance of inflammatory and nutritional biomarkers in predicting SWIs. The corresponding ROC curves for deep SWIs are presented in Figure 3A–F.

Preoperative NLR demonstrated excellent accuracy for identifying deep infections, with an AUC of 0.880 (95% CI: 0.82–0.93; *p* < 0.001) and an optimal cutoff of 3.75, yielding 94.4% sensitivity and 75.4% specificity. It also retained substantial predictive capacity for superficial SWIs (AUC = 0.768; 95% CI: 0.74–0.86; *p* < 0.001), with a threshold of 3.1. In contrast, preoperative CAR yielded only moderate discriminative ability for deep infections (AUC = 0.760; 95% CI: 0.64–0.89; *p* < 0.001; cutoff: 3.2), and its performance was notably limited for superficial SWIs (AUC = 0.634; *p* = 0.001).

Preoperative PNI showed fair diagnostic value for predicting deep SWIs (AUC = 0.731; 95% CI: 0.60–0.89; *p* < 0.001), with a cutoff value of 39.5. However, its performance in identifying superficial infections was modest (AUC = 0.584; 95% CI: 0.51–0.65; *p* = 0.021), suggesting limited utility in this subgroup. Despite this, the consistent association between reduced PNI values and deep infections highlights its potential role in preoperative risk stratification, particularly for nutritionally vulnerable patients.

To assess early postoperative inflammatory changes, delta-based ROC analyses were also performed. ΔNLR demonstrated strong predictive performance for deep SWIs (AUC = 0.841; 95% CI: 0.77–0.90; *p* < 0.001; cutoff: 9.8). Notably, ΔCAR exhibited the highest overall diagnostic accuracy (AUC = 0.895; 95% CI: 0.80–0.99; *p* < 0.001; cutoff: 16.7), offering excellent sensitivity and specificity. This marked elevation in the CAR may reflect the combined effects of acute-phase inflammation and early postoperative nutritional deterioration. In summary, while preoperative NLR was the most reliable baseline biomarker, ΔCAR proved to be the most robust early postoperative indicator for identifying patients at elevated risk of deep SWIs.

## 4. Discussion

This study provides compelling evidence that the combined use of intraoperative sternal irrigation and a locally applied antibiotic-impregnated membrane significantly reduces early postoperative systemic inflammation and the incidence of both superficial and deep SWIs following OPCABG. Patients receiving this targeted intervention exhibited more favorable inflammatory and nutritional profiles, including lower CRP levels, reduced NLR and CAR values, and preserved albumin and PNI levels. Despite a 2.1-fold increased risk of SWIs associated with prolonged operative durations, this approach was notably linked to a 1.1-fold reduction in superficial SWIs and an approximately 1.8-fold decrease in deep SWIs, highlighting its dual benefit in mitigating both minor and severe infectious complications. Preoperative NLR, in particular, demonstrated excellent diagnostic accuracy for predicting deep SWIs, further reinforcing its clinical relevance in early risk stratification. Moreover, dynamic postoperative changes in CRP, ΔNLR, and ΔCAR were more pronounced in patients who developed deep infections, supporting their potential utility as early indicators of adverse outcomes. These findings highlight the value of integrating localized antimicrobial strategies with biomarker-driven surveillance to optimize postoperative infection control in cardiac surgery.

SWIs, typically classified as either superficial or deep, occur in up to 10% of patients undergoing cardiac surgery. Although relatively infrequent, deep infections—particularly mediastinitis—carry a mortality risk of up to 50%, representing one of the most feared complications in the postoperative setting [27,28]. Beyond their direct impact on survival, SWIs are associated with a wide array of adverse outcomes, including prolonged hospital stay, increased healthcare expenditures, diminished long-term survival, and substantial psychological distress due to persistent wound discharge and the need for repeated surgical debridement [29]. While identification of high-risk individuals and the management of modifiable risk factors remain essential, the implementation of effective and standardized preventive strategies is of paramount importance in contemporary cardiac surgical practice [5,30].

In cardiac surgery, every anatomical layer—from the skin to the mediastinum—is susceptible to infection, and the clinical spectrum can range from minor serous drainage to catastrophic mediastinitis accompanied by extensive necrosis and sternal osteomyelitis [31,32,33]. Within this context, minimizing the risk of wound-related complications necessitates a comprehensive, multidisciplinary approach that includes meticulous intraoperative conduct by the surgical team, an optimized operating room environment, and refined surgical techniques [34]. Optimal preoperative control of modifiable risk factors such as obesity and diabetes, along with proper subcutaneous tissue handling and layered wound closure, is essential to reduce superficial SWI incidence [35]. Residual dead spaces and electrocautery-induced fat necrosis can facilitate bacterial growth and adherence to sutures [36,37]. Thus, irrigating subcutaneous tissues and skin—especially with antibiotic solutions—may help lower microbial load and prevent superficial infections [38].

Minimizing airborne bacterial contamination is a key intraoperative strategy, achievable through limiting personnel and movement in the operating room and maintaining controlled ventilation systems [10,39]. This is especially important for reducing the aerosolization of skin flora—mainly Staphylococcus spp.—which are leading pathogens in deep SWIs [40]. Yavuz et al. [41] associated outdated OR environments and operative times > 5 h with a higher SWI risk, while Kühme et al. [42] identified both commensal and nosocomial bacteria in sternal wounds, regardless of duration. In our cohort, operative time was significantly longer in cases of deep SWI. Notably, a ROC-defined threshold of 108 min indicated that each additional 15 min increase raised the risk of deep infection 2.1-fold. Although incisional films were routinely applied to reduce airborne exposure, it is important to acknowledge that an open mediastinum remains susceptible to both intrinsic and extrinsic contamination—particularly during prolonged surgical interventions.

Another important strategy in reducing intraoperative contamination involves the use of sternal local antibiotic application, which has emerged as a promising prophylactic approach for the prevention of SWIs and is increasingly being adopted by cardiac surgery centers worldwide [29]. However, a universally accepted consensus regarding its routine use, optimal antibiotic formulation, and standardized implementation has yet to be established. While certain clinical guidelines advise against the routine use of local antibiotic prophylaxis in sternal wound management [43], a meta-analysis by López-Cano et al. [44] reported a significant reduction in surgical site infections (RR ≈ 0.49; 95% CI: 0.37–0.64) with topical antibiotic application. Despite this favorable outcome, the authors highlighted limitations in evidence quality and consistency, emphasizing the need for more robust data before endorsing routine clinical use. In addition, other studies have drawn attention to the marked heterogeneity in clinical practice—particularly regarding the selection of local agents such as gentamicin, bacitracin, or vancomycin—and to the multifactorial nature of SWIs, both of which complicate the establishment of standardized protocols [45,46,47].

Due to the limited strength of evidence regarding topical antibiotic use, significant variability in clinical practice, and even the existence of guidelines that do not support their routine application, this approach has yet to achieve universal acceptance or a standardized guideline recommendation [43,48,49]. Despite differing opinions on the use of topical antibiotics, the 2016 guideline issued by the American Association for Thoracic Surgery (AATS) [25] provides a clear recommendation: “Topical antibiotics should be applied to the cut edges of the sternum on opening and before closing in all cardiac surgical procedures involving a median sternotomy” (Class I Recommendation; Level of Evidence = B). The guideline specifically endorses the use of vancomycin as the preferred topical agent in this context. A recent meta-analysis demonstrated a 69% reduction in both superficial and deep SWIs with topical vancomycin (RR: 0.31; 95% CI: 0.23–0.43; *p* < 0.001), with particularly enhanced efficacy in diabetic patients and a notable decrease in Gram-negative pathogens [50]. Vancomycin paste has emerged as a practical prophylactic option—offering ease of application, cost-effectiveness, and low systemic toxicity. Its association with deep SWI rates falling below 1% further highlights its clinical relevance [51,52,53].

However, some studies have reported that topical vancomycin application did not result in a significant reduction in SWI rates [46,54]. Although these divergent findings may be influenced by variations in patient populations and application techniques, the overall body of evidence supports the efficacy of topical antibiotics—whether vancomycin or gentamicin—in decreasing the incidence of both deep (RR: 0.60; 95% CI: 0.43–0.83; *p* = 0.003) and superficial SWIs (RR: 0.54; 95% CI: 0.32–0.91; *p* = 0.02) [55].

In our clinical practice, we employ a fascia lata-derived membrane soaked for 10 min in 500 mL of 0.9% saline solution containing 240 mg of gentamicin [56]. After positioning the membrane along the sternal surface, the remaining antibiotic-enriched solution is used to irrigate the surgical field before closure of the subcutaneous tissue and skin. Beyond the structural support provided by collagen, the local antimicrobial action of gentamicin—augmented by intraoperative irrigation—appears to play a pivotal role in infection prevention.

Locally administered gentamicin can achieve high antimicrobial concentrations at the site of application without elevating systemic serum levels, thereby minimizing the risk of toxicity. Moreover, by maintaining sustained high concentrations at the local site—particularly on implanted materials—it can inhibit bacterial adhesion and biofilm formation, even against pathogens typically considered resistant under systemic treatment conditions [57,58]. However, concerns regarding systemic absorption and resistance development have prompted recommendations for its cautious use [59]. Friberg et al. [60] reported that the use of local collagen–gentamicin antibiotic prophylaxis remained consistently effective over approximately seven years of routine clinical application, with no observed decline in efficacy. Importantly, there was no significant increase in SWIs caused by aminoglycoside-resistant microorganisms, nor any notable shift in the spectrum of causative pathogens. The majority of infections continued to be attributed to coagulase-negative staphylococci. Although gentamicin resistance was not observed, the addition of agents with enhanced Gram-positive coverage to gentamicin-based prophylaxis may further reduce the incidence of SWIs [55].

Since implementing this combined approach, we have observed a significant reduction in the incidence of SWIs. Specifically, both superficial and deep infections were markedly less frequent in the local antibiotic group compared to the control group (4 vs. 14 deep and 26 vs. 49 superficial SWIs; *p* = 0.001) [8,61,62]. Our findings align with those reported by Friberg [63], who demonstrated that local antibiotic application was associated with an approximate 50% reduction in overall SWI risk. Similarly, Osawa et al. [64] observed a decreased incidence of deep SWIs in patients receiving intraoperative antibiotic spraying directly onto the surgical field, suggesting an additional layer of protection during critical procedural phases. Together, these observations support the clinical value of locally delivered antibiotics as a practical and effective adjunct in SWI prevention strategies. On the other hand, the overall SWI rate in our cohort—19.4% (93 out of 480 patients)—appears somewhat higher than the average rates reported in the literature. However, it is important to emphasize that most published studies primarily focus on deep SWIs, whereas our reported incidence reflects a cumulative value encompassing both deep and clinically significant superficial infections. Specifically, we also included non-healing superficial wounds characterized by fat necrosis, persistent drainage, or fistula formation—conditions that, while not strictly fulfilling the diagnostic criteria for deep SWIs, still posed considerable clinical challenges and necessitated prolonged follow-up and treatment [3,23]. Given the high-risk profile of our patient population—particularly the prevalence of diabetes, obesity, and advanced age—this rate may be better contextualized within the spectrum of outcomes observed in complex cardiac surgical cohorts [5].

The variability in reported incidence rates across studies may also reflect differences in classification systems, clinical thresholds, and institutional practices regarding the diagnosis and documentation of SWIs [65]. Nevertheless, the incidence of SWIs unfortunately remains above zero in most centers, despite the widespread adoption of various preventive strategies. Notably, Downing et al. [66] reported achieving a zero percent annual infection rate through their “I Hate Infections Team” initiative—a result likely driven not only by strict institutional protocols, but also by targeted efforts to mitigate the postoperative inflammatory burden beyond conventional surgical risk factors.

In parallel, our findings suggest that the reduced incidence of SWIs in the sternal intervention group may be partly explained by a less intense systemic inflammatory response, as evidenced by consistently lower NLR and CAR values and higher PNI scores. Patients who developed deep SWIs exhibited significantly elevated inflammatory indices, underscoring the role of postoperative inflammation in infection pathogenesis [67]. This observation is supported by Madjarov et al. [68], who reported persistently elevated inflammatory markers at discharge among patients with SWIs.

Given the invasive nature of CABG and its capacity to trigger robust immune activation [69], a heightened inflammatory response is not unexpected. Furthermore, foreign materials such as sternal closure devices and microbial contaminants can amplify this response. In an experimental model, Ersöz et al. [70] demonstrated that stainless steel implants in the presence of bacterial suspensions resulted in pronounced neutrophil activation and oxidative stress.

The fascia lata-derived membrane combined with gentamicin irrigation may exert a protective effect by forming a localized barrier that limits proinflammatory triggers and reinforces host defenses [71]. This effect may also involve surface modifications that reduce bacterial adhesion, particularly by disrupting interactions between fibronectin-binding proteins of Staphylococcus aureus and host or prosthetic surfaces [6,72,73].

The persistently lower postoperative levels of inflammatory markers—especially the CAR and NLR—observed in the intervention group support the hypothesis that this strategy contributes not only to local infection control but also to attenuation of the systemic inflammatory response [74,75].

Common comorbidities in patients with coronary artery disease—such as diabetes mellitus, obesity, and smoking—are well-recognized risk factors for SWIs [76,77]. To address the clinical complexity of SWIs, various risk scoring systems have been developed to identify high-risk patients; however, their predictive accuracy remains inconsistent across surgical populations [78]. Importantly, most of these models do not incorporate inflammatory biomarkers, despite inflammation being central to SWI pathogenesis. In a histopathological study of resected sternal bone from 47 patients with confirmed SWIs, Bota et al. [79] found inflammatory cell infiltration in 76.6% to 93.6% of samples, highlighting the ubiquity of inflammation in these infections.

Among emerging biomarkers, the NLR stands out as a simple yet informative composite marker that captures both innate (neutrophil-driven) and adaptive (lymphocyte-mediated) immune responses. Its prognostic relevance has been widely studied across various cardiovascular surgical settings [80,81]. For instance, Mao et al. [82] demonstrated significantly elevated NLR levels (*p* = 0.003) in cardiac surgery patients who developed postoperative infections; however, their analysis did not differentiate between infection subtypes. In contrast, Parla et al. [16] specifically explored the predictive value of the NLR in deep SWIs—a clinically valuable focus, given the limited number of studies directly linking the NLR to sternal wound complications. Measuring inflammatory markers on postoperative days 1 and 2, they reported elevated NLR levels in patients who developed deep infections. On postoperative day 1, an optimal NLR cutoff of 11.2 yielded modest discriminative accuracy (AUC: 0.598; *p* = 0.014; sensitivity: 60.0%, specificity: 65.2%), while the delta NLR threshold of 9.6 showed improved performance (AUC: 0.716; *p* < 0.001; sensitivity: 57.1%, specificity: 73.8%).

Unlike the approach adopted by Parla et al. [16], our study included only patients undergoing off-pump CABG to eliminate the confounding inflammatory effects of cardiopulmonary bypass and ensure a more homogeneous surgical cohort. To refine the prediction of SWI development, we evaluated both baseline and dynamic markers that could be independent predictors. Among all biomarkers assessed, preoperative NLR showed the highest predictive accuracy for deep SWIs, with an AUC of 0.880 (95% CI: 0.82–0.93; *p* < 0.001); a threshold of 3.75 provided 94.4% sensitivity and 75.4% specificity. ΔNLR also demonstrated strong discriminatory capacity (AUC = 0.841; 95% CI: 0.77–0.90) with a cutoff of 9.8. Similarly, ΔCAR exhibited excellent predictive performance (AUC = 0.895; 95% CI: 0.80–0.99; *p* < 0.001), with a threshold of 16.7 yielding 93.2% sensitivity and 81.2% specificity. These findings underscore the clinical relevance of monitoring temporal fluctuations in inflammatory indices for early detection of patients at elevated risk of deep SWIs.

In cardiac surgery patients, the underlying proinflammatory state—exacerbated by surgical stress—can blunt the immune response to postoperative infections, delaying their recognition. In this setting, sustained elevations of biomarkers such as CRP, whose postoperative kinetics are well characterized, may aid in distinguishing pathological infections from expected inflammatory responses [83]. However, the early postoperative rise in CRP, often sustained through the third postoperative day, can reduce its diagnostic specificity during this period [84,85]. Foldyna et al. [86] reported that although CRP levels remained elevated in mediastinitis, its discriminatory ability was only moderate (AUC = 0.73; 95% CI, 0.59–0.86), and accordingly, they recommended combining CRP assessment with computed tomography to improve diagnostic accuracy.

Jia et al. [87] evaluated serial inflammatory markers at five distinct postoperative time points in a cohort of cardiac surgery patients, including 30 individuals who developed deep SWIs and 211 without deep SWIs. The authors reported that, starting from postoperative day 7, inflammatory marker levels were significantly higher in patients who developed deep SWIs. In particular, they highlighted the diagnostic value of CRP on postoperative days 10 and 14, with reported AUC values of 0.786 (95% CI: 0.699–0.872; cutoff: 170.2 mg/L) and 0.800 (95% CI: 0.723–0.878; cutoff: 64.4 mg/L), respectively. Based on these findings, the authors suggested that CRP may serve as a more reliable biomarker than the NLR in the late postoperative period.

Unlike the study conducted by Jia et al. [87], our investigation focused not only on preoperative and postoperative (day 3 and week 1) inflammatory data but also on the dynamic changes from baseline—i.e., the delta values. In our cohort, preoperative CRP levels were already elevated in patients who developed deep SWIs and independently predicted deep infection risk (OR: 2.2; 95% CI: 1.4–2.9; *p* = 0.007). However, given the complex interplay between inflammation and nutritional status—as well as CRP’s known susceptibility to multiple regulatory influences including gene polymorphisms, interleukin signaling, and other physiological factors—our findings support the rationale for using CRP in combination with additional biomarkers to enhance diagnostic accuracy [88,89]. Furthermore, in such patients, prolonged treatment of drug-resistant organisms—typically nosocomial pathogens—may contribute to persistent or fluctuating CRP levels, thereby complicating the interpretation of inflammatory status [90]. In contrast to CRP, which rises rapidly in response to tissue injury as part of the acute-phase reaction, the NLR reflects both innate and adaptive components of the immune system, making it a more specific and sustained indicator of the patient’s immunoinflammatory status [16]. Nevertheless, given their interrelation, the combined assessment of CRP and the NLR highlights their potential clinical value in improving diagnostic precision and postoperative risk stratification [91].

Sternal wound healing is anatomically distinct from that of other surgical sites, as it requires the coordinated regeneration of multiple tissue layers, including bone, muscle, and subcutaneous tissue. This complexity—combined with the physiological stress of cardiac surgery and the frequent presence of systemic comorbidities—places sternal incisions in a unique risk category [92,93]. Beyond the influence of inflammatory responses, the patient’s nutritional status plays a critical role in maintaining immune competence. Major surgical stress, often exacerbated by postoperative gastrointestinal dysfunction, may precipitate or worsen malnutrition, thereby increasing susceptibility to infection. Lazar [94] has recommended deferring elective procedures in malnourished patients to allow for preoperative nutritional optimization. Supporting this, Bae et al. [18] observed a lower incidence of SWIs among patients with improved PNI scores. Consistent with these findings, our analysis showed that patients who developed SWIs exhibited persistently lower serum albumin and PNI levels throughout hospitalization and early postoperative follow-up. Moreover, these nutritional indicators declined progressively in accordance with infection severity. In this context, the CAR—which reflects the interplay between inflammation and nutritional reserve—may offer additional insight into the systemic inflammatory load, particularly in hypoalbuminemic patients.

The predictive utility of biomarkers and risk stratification systems enables early identification of postoperative complications—such as infection and sternal dehiscence—in patients undergoing sternotomy. This facilitates more vigilant, individualized, and timely preventive strategies, thereby enhancing patient safety, improving surgical outcomes, and potentially reducing the overall healthcare burden [95,96]. In the United States, the management of SWIs following coronary artery bypass grafting can exceed USD 60,000 per patient, and in Türkiye, deep SWIs have been reported to cost approximately twice as much as superficial infections [97,98]. Friberg et al. [99] demonstrated that local collagen–gentamicin application reduced infection rates and was cost-effective, whereas Joshi et al. [100] cautioned that routine use in all high-risk patients may not be economically justified given the product’s cost. ’Although our study was not primarily designed to assess cost-effectiveness, retrospective hospitalization data revealed that the average cost per patient was TRY 69,565 in the membrane group and TRY 65,040 in the control group. Despite the higher cost associated with membrane use, the marked reduction in deep SWI cases (from 14 to 4) suggests a potential economic benefit, likely stemming from reduced complication-related expenditures. Given the high resource utilization associated with deep infections, such prophylactic strategies may offer long-term savings. However, due to variability in healthcare systems and cost structures, prospective economic evaluations are warranted to confirm these findings [30].

## 5. Limitations

This study has several limitations. Its retrospective, single-center design may limit generalizability, and unmeasured confounding variables cannot be entirely excluded despite a sufficient sample size. The temporal separation between the control and intervention groups—due to protocol changes implemented in 2021—may have introduced time-related confounding, such as evolving perioperative practices, antimicrobial resistance trends, or institutional infection control measures. Although limiting the cohort to off-pump CABG patients ensures homogeneity, it may reduce applicability to broader cardiac surgery populations. The local antibiotic protocol, based on institutional experience, warrants validation in larger, multicenter cohorts—especially for its efficacy against isolated deep SWIs. The relatively small number of deep SWI cases (*n* = 18), while clinically favorable, may have limited statistical power and introduced bias in subgroup analyses. To address this, we employed robust logistic regression with 5000 bootstrap iterations; however, external validation using real-world data is still needed. Furthermore, the gentamicin used in the impregnated membrane primarily targets Gram-negative bacteria and offers limited coverage against broader Gram-positive flora. Future studies should evaluate broader-spectrum or combination agents, particularly in high-risk or immunocompromised patients [62]. Finally, while inflammatory and nutritional biomarkers were assessed perioperatively, longer-term trajectories were not analyzed. Indices such as the NLR, CAR, and PNI, although practical and low-cost, are non-specific and may be influenced by concurrent comorbidities, requiring cautious interpretation in diverse clinical contexts [101].

## 6. Conclusions

The implementation of local sternal interventions—namely antibiotic-impregnated membranes combined with intraoperative irrigation—emerged as a key strategy in reducing early postoperative inflammation and the incidence of SWIs. Patients who received this prophylactic approach exhibited significantly lower rates of both superficial and deep SWIs, along with more favorable inflammatory and nutritional profiles. Among all evaluated biomarkers, preoperative NLR and ΔCAR showed the highest predictive accuracy for deep infections, underscoring their value in early risk stratification. These findings support the integration of simple, cost-effective indices into perioperative monitoring and emphasize the clinical utility of targeted local prophylaxis in improving surgical outcomes. Prospective, multicenter studies are needed to validate these results and to develop refined risk models that incorporate both inflammatory and nutritional markers.

## Figures and Tables

**Table 1:** Formulas and calculation methods of indices used in study [20].

Index/Parameter	Calculation Method
Body mass index (kg/m^2^) (BMI)	Weight (kg)/Height (m^2^)
C-reactive protein-to-albumin ratio (CAR)	CRP (mg/L)/Albumin (g/dL)
Neutrophil-to-lymphocyte ratio (NLR)	Neutrophil count/Lymphocyte count
Prognostic nutritional index (PNI)	[10 × serum Albumin (g/dL)] + [0.005 × Lymphocyte count]
Delta value (Δ)	Postoperative day 3 minus preoperative measurements

**Table 2:** Preoperative demographic and clinical parameters between control and sternal intervention groups.

**Patient Demographics**	Control Group (*n* = 250)	Sternal Intervention Group * (*n* = 230)	*p*
Female/male	54/196	69/161	0.065
Age (years)	61.5 ± 8.5	62.4 ± 7.9	0.438
Body mass index, (kg/m^2^)	28.1 ± 3.0	27.7 ± 3.0	0.124
EuroSCORE	1.5 ± 0.9	1.6 ± 0.8	0.107
Ejection fraction, %	51.0 ± 6.0	51.2 ± 6.9	0.312
Diabetes mellitus, *n* (%)	105 (42)	103 (44.7)	0.539
Hypertension, *n* (%)	113 (45.2)	106 (46.0)	0.845
Obesity (BMI ≥ 30 kg/m^2^), *n* (%)	63 (25.2)	55 (24.0)	0.744
Current smoker, *n* (%)	104 (41.6)	82 (35.6)	0.181
Cerebrovascular event, *n* (%)	14 (5.6)	12 (5.2)	0.853
Peripheral artery disease, *n* (%)	20 (8.0)	27 (11.7)	0.169
COPD, *n* (%)	51 (20.4)	55 (23.9)	0.354
Glucose, mg/dL	157 ± 61	156 ± 64	0.866
Cr, mg/dL	1.0 ± 0.6	0.9 ± 0.2	0.142
Hemoglobin, g/dL	14.8 ± 1.3	14.8 ± 1.4	0.701
^a^ Neutrophil count, 10^3^/μL, median (Q1–Q3)	6.1 (4.9–7.1)	5.9 (4.8–6.6)	0.410
^a^ Lymphocyte count, 10^3^/μL, median (Q1–Q3)	2.0 (1.5–2.3)	2.1 (1.5–2.4)	0.334
^a^ Monocyte count, 10^3^/μL, median (Q1–Q3)	0.63 (0.53–0.78)	0.65 (0.55–0.79)	0.403
Platelet count, 10^3^/μL	253 ± 60	257 ± 50	0.155
LDL, mg/dL	115 ± 30	110 ± 27	0.744
Triglyceride, mg/dL	159 ± 62	150 ± 48	0.370
Albumin, g/dL	4.0 ± 0.3	4.2 ± 0.3	0.040
C-reactive protein, mg/L	7.2 ± 2.3	6.9 ± 1.9	0.337
HbA1c, %	6.7 ± 1.9	6.5 ± 1.8	0.203
^b^ NLR; median (Q1–Q3)	2.9 (2.3–4.1)	2.8 (2.3–3.6)	0.575
^b^ CAR, median (Q1–Q3)	1.7 (1.4–2)	1.6 (1.3–1.9)	0.241
^b^ PNI, median (Q1–Q3)	42 (39.6–43.5)	43 (40.8–44.2)	0.020

BMI, body mass index; CAR, C-reactive protein-to-albumin ratio; COPD, chronic obstructive pulmonary disease; Cr, creatinine; HbA1c, glycated hemoglobin; LDL, low-density lipoprotein; NLR, neutrophil-to-lymphocyte ratio; PNI, prognostic nutritional index. * Sternal intervention group: irrigation + antibiotic membrane; a, Mann–Whitney U test; b, Brunner–Munzel test.

**Table 3:** Comparative temporal trends of inflammatory and nutritional markers between groups.

Parameters	Control Group (*n* = 250)			Sternal Intervention Group * (*n* = 230)			
	Preoperative	Postoperative Day 3	Postoperative Week 1	Preoperative	Postoperative Day 3	Postoperative Week 1	*p* *
CAR, median (Q1–Q3)	1.7 (1.4–2)	32 (25–40)	4.2 (2.5–7.7)	1.6 (1.3–1.9)	25 (21–32)	3.3 (2.4–5.1)	<0.001
NLR; median (Q1–Q3)	2.9 (2.3–4.1)	11 (8.6—14.6)	7.2 (5.0–11.2)	2.8 (2.3–3.6)	7.8 (5.9–10.7)	4.2 (3.3–5.8)	<0.001
PNI, median (Q1–Q3)	42 (39.6–43.5)	38 (33.0–40.2)	39 (36.6–41.7)	43 (40.8–44.2)	40 (36.9–41.4)	41.4 (39.0–42.5)	<0.001
Albumin, g/dL, median (Q1–Q3)	4.2 (4.0–4.3)	3.8 (3.3–4.0)	3.9 (3.7–4.2)	4.3 (4.0–4.5)	4.0 (3.7–4.2)	4.2 (3.9–4.3)	<0.001
CRP, mg/L median (Q1–Q3)	7.1 (5.7–8.1)	120 (96–141)	16.2 (10.9–28.3)	6.8 (5.5–8.1)	101 (83–120)	12.8 (9.6–19.0)	<0.001
Nc, 10^3^/μL, median (Q1–Q3)	6.1 (4.9–7.1)	12.8 (11.2–14.5)	8.8 (7.7–10.5)	5.9 (4.8–6.6)	11.1 (9.5–13.0)	7.6 (6.8–8.6)	<0.001
Lc, 10^3^/μL median (Q1–Q3)	2.0 (1.5–2.3)	1.1 (0.9–1.4)	1.2 (0.9–1.6)	2.1 (1.5–2.4)	1.4 (1.1–1.9)	1.9 (1.4–2.3)	<0.001

CAR, C-reactive protein-to-albumin ratio; CRP, C-reactive protein; Lc, lymphocyte count; Nc, neutrophil count; NLR, neutrophil-to-lymphocyte ratio; PNI, prognostic nutritional index. * *p*-values indicate intergroup differences in biomarker changes over the three time points assessed using repeated measures ANOVA or the Friedman test, depending on data distribution. * Sternal intervention group: irrigation + antibiotic membrane.

**Table 4:** Clinical and demographic characteristics of patients stratified by severity of sternal wound infections.

**Patient Demographics**		Superficial SWIs (*n* = 75)	Deep SWIs (*n* = 18)	*p*
Female/male		22/53	10/8	0.012
Age (years)		62.9 ± 8.0	64.8 ± 7.6	0.369
Body mass index, (kg/m^2^)		28.8 ± 2.8	33.4 ± 3.9	<0.001
EuroSCORE		1.8 ± 1.0	2.4 ± 1.0	0.023
Ejection fraction, %		49.0 ± 5.9	49.2 ± 5.6	0.925
Diabetes mellitus, *n* (%)		55 (73)	16 (89)	0.137
Hypertension, *n* (%)		42 (56)	12 (66)	0.410
Obesity (BMI ≥ 30 kg/m^2^), *n* (%)		30 (40)	15 (83)	0.001
Current smoker, *n* (%)		49 (65)	7 (39)	0.040
Cerebrovascular event, *n* (%)		3 (4)	4 (22)	0.024
Peripheral artery disease, *n* (%)		12 (16)	2 (11)	0.461
COPD, *n* (%)		21 (28)	10 (55)	0.026
Nc, 10^3^/μL, median (Q1–Q3)		6.5 (5.4–7.3)	8.0 (7.4–8.8)	<0.001
Lc, 10^3^/μL median (Q1–Q3)		1.8 (1.3–2.1)	1.5 (1.0–2.0)	0.150
LDL, mg/dL		120 ± 30	142 ± 26	0.010
Triglyceride, mg/dL		170 ± 56	195 ± 58	0.112
Albumin, g/dL		4.0 ± 0.3	3.7 ± 0.4	0.001
CRP, mg/L median (Q1–Q3)		7.6 (6.0–8.5)	8.3 (6.7–9.5)	0.016
HbA1c, %		7.6 ± 1.9	10.2 ± 2.7	0.001
NLR; median (Q1-Q3)		3.5 (2.9–5.2)	4.7 (4.0–7.2)	0.005
CAR, median (Q1–Q3)		1.8 (1.5–2.1)	2.2 (1.6–2.6)	0.075
PNI, median (Q1–Q3)		40.9 (38.6–42.2)	38.6 (32.6–40.6)	0.002
ΔNLR ** median (Q1–Q3)		6.9 (4.9–9.7)	11.5 (8.2–15.8)	0.003
ΔCAR ** median (Q1–Q3)		34.5 (30.4–41.1)	61.6 (52.2–76.0)	<0.001
ΔPNI ** median (Q1–Q3)		−4.8 (−8.5–−3.0)	−5.5 (−6.8–−2.9)	0.985
Distal anastomoses (mean ± SD)		2.8 ± 0.7	3.0 ± 0.8	0.077
Graft types, *n* (%)	LIMA	75 (100)	18 (100)	1.000
	Radial artery	26 (35%)	7 (39)	0.787
	Saphenous vein	70 (93%)	16 (89)	0.617
Operative time, minutes		100 ± 25	135 ± 30	<0.001
Red blood cell transfusion, units		1.2 ± 0.6	1.3 ± 0.8	0.095

CAR, C-reactive protein-to-albumin ratio; CRP, C-reactive protein; COPD, chronic obstructive pulmonary disease; HbA1c, glycated hemoglobin; Lc, lymphocyte count; LDL, low-density lipoprotein; LIMA, left internal mammary artery; Nc, neutrophil count; NLR, neutrophil-to-lymphocyte ratio; PNI, prognostic nutritional index, SWIs, sternal wound infections. ** Δ (Delta) values represent the change from preoperative measurements to postoperative day 3 (i.e., postoperative day 3 minus preoperative).

**Table 5:** Multinomial regression analysis of predictors for superficial and deep sternal wound infections.

Predictor	Superficial SWIs		Deep SWIs	
	OR (95% CI)	*p*	OR (95% CI)	*p*
Sternal intervention *	0.89 (0.5–1.3)	0.061	0.55 (0.15–0.87)	0.001
Prolonged operative time (>108 min)	1.1 (0.91–1.9)	0.095	2.1 (1.2–3.1)	0.012
Female	2.9 (1.2–7.9)	0.027	3.8 (1.7–10.2)	0.003
Age, years	1.1 (1.01–1.5)	0.039	1.3 (1.1–3.4)	0.012
Diabetes mellitus	2.8 (1.3–8.8)	<0.001	3.9 (1.6–9.8)	<0.001
Current smoking	2.1 (1.5–8.7)	0.004	3.8 (1.3–9.9)	<0.001
Chronic obstructive pulmonary disease	1.3 (0.6–5.2)	0.328	1.9 (0.76–7.1)	0.122
HbA1c (≥7%)	1.5 (1.1–2.0)	0.011	3.1 (1.6–5.9)	<0.001
C-reactive protein	1.4 (0.71–3.3)	0.076	2.2 (1.4–2.9)	0.007
Albumin (≥3.8 g/dL)	0.9 (0.15–0.81)	0.004	0.53 (0.2–0.93)	<0.001
Neutrophil-to-lymphocyte ratio	1.5 (1.2–2.4)	0.002	2.2 (1.3–4.8)	0.001
* Delta neutrophil-to-lymphocyte ratio	1.06 (0.9–1.1)	0.253	1.2 (1.1–3.9)	0.003
C-reactive protein-to-albumin ratio	1.2 (0.71–3.0)	0.751	2.9 (0.68–5.6)	0.220
* Delta C-reactive protein-to-albumin ratio	1.1 (1.0–1.2)	0.034	2.4 (1.3–5.6)	<0.001
Prognostic nutritional index	0.72 (0.4–0.95)	0.001	0.52 (0.31–0.90)	0.004

HbA1c, glycated hemoglobin; * sternal intervention group: irrigation + antibiotic membrane; * Δ (delta) values represent the change from preoperative measurements to postoperative day 3 (i.e., postoperative day 3 minus preoperative).

## Data Availability

All data generated or analyzed during this study, including imaging materials, laboratory findings, and statistical data, are securely stored within the authors’ archives. No data were obtained from external sources or previously published materials. These datasets are available from the corresponding author upon reasonable request.

## Abbreviations

AUC	Area under the curve
BMI	Body mass index
CAR	C-reactive protein-to-albumin ratio
COPD	Chronic obstructive pulmonary disease
CRP	C-reactive protein
NLR	Neutrophil-to-lymphocyte ratio
OPCABG	Off-pump coronary artery bypass grafting
OR	Odds ratio
PNI	Prognostic nutritional index
ROC	Receiver operating characteristic
Δ	Delta
